# Phenotypic and genetic characterisation revealed the existence of several biotypes within the *Neorautanenia brachypus* (Harms) C.A. wild accessions in South East Lowveld, Zimbabwe

**DOI:** 10.1186/s12898-019-0229-9

**Published:** 2019-03-12

**Authors:** Trish. O. Nyarumbu, Tinotenda Kaseke, Vimbai Gobvu, Chrispen Murungweni, Arnold. B. Mashingaidze, Zedias Chikwambi

**Affiliations:** 1 Horticulture Research Institute, P.O. Box 810, Marondera, Zimbabwe; 2 Department of Biotechnology, Chinhoyi University of Technology, P. Bag 7724, Chinhoyi, Zimbabwe; 3 Department of Animal Production and Technology, Chinhoyi University of Technology, P. Bag 7724, Chinhoyi, Zimbabwe; 4 Department of Crop Science and Post-Harvest Technology, Chinhoyi University of Technology, P. Bag 7724, Chinhoyi, Zimbabwe

**Keywords:** Tuber, Random amplified polymorphic DNA (RAPD), Primer, Binary coding, Dendrogram, Genetic variability

## Abstract

**Background:**

Local communities in the South Eastern Lowveld of Zimbabwe have adopted the feeding of livestock with *Neorautanenia brachypus* (Harms) C.A. tuber to mitigate against climate change. Differences within *Neorautanenia brachypus* (Harms) tuber flesh colour and preferences by cattle have been observed, suggesting possible diversity within the *N. brachypus* plant community. This study aimed at distinguishing the *N. brachypus* wild plant species through phenotypic and genetic characterization using morphological descriptors and random amplified polymorphic (RAPD) markers respectively. Leaf samples were selected using judgmental sampling techniques from wards 11–15 in Sengwe (Chiredzi district) for leaf morphology and molecular characterization. RAPD-PCR analysis was done using 18-screened random decamer primers to confirm the diversity in the plant population. The similarity of the biotypes was evaluated using binary coding on the basis of the presence or absence of a morphological indicator as well as distinct DNA amplicon fragments. Primer 7.0.13 was used to estimate morphological and genetic similarities using the unweighted pair group method with arithmetic average (UPGMA). The cluster number was estimated using the Elbow method part of the R package.

**Results:**

Initially, 14 biotype groups were identified from 96 accessions visually characterized basing of leaf characteristics. All the leaf biotypes displayed arcuate venation with differences observed for leaf shape, tip shape and leaf margins. The 14 biotypes clustered into six groups based on the binary data of the morphological characteristics. RAPD primers generated three hundred and sixty eight distinct amplicons with 77.5% being polymorphic from the 14 biotypes. The number of bands produced per primer ranged from four (OPF-02) to 44 (UBC-746). The PIC value ranged from 0.1327 to 0.1873 for the RAPD primers. Use of molecular markers collapsed the biotypes into five clusters. Both the leaf descriptors and RAPD markers showed the existence of genetic diversity within the wild accessions of *N. brachypus*.

**Conclusions:**

A combination of morphological and RAPD markers effectively refined the resolution of the genetic diversity within the *N. brachypus* wild accessions to nine biotypes. These findings have indicated to the existence of more than one biotype of *N. brachypus* with potentially different properties. The favorable biotypes can further be promoted through incorporation in pastures as alternative feed or complementary feed to livestock. As such the output of this study will serve as a guide for *N. brachypus* germplasm management and improvement.

**Electronic supplementary material:**

The online version of this article (10.1186/s12898-019-0229-9) contains supplementary material, which is available to authorized users.

## Background

Recently, *Neorautanenia brachypus* (Harms) C.A. was identified in the South Eastern Lowveld of Zimbabwe as an important multipurpose legume tuber plant. The plant has been used as an alternative ruminant animal feed during periods of drought, wound remedy for livestock and a botanical pesticide against internal parasites in ruminants [1]. It belongs to the Leguminosae-Papilionaceae family. *N. brachypus* produces purple flowers, which forms dehiscent pods densely covered by hairs [2]. The chemical composition and nutritional values of the tubers reported by [1], indicated that the tubers can be used as a sole feed for cattle. Livestock play an important role in the household economies of families in arid regions of Zimbabwe [3, 4] and the worsening climatic conditions will be a huge challenge to livestock production. Farmers will be hard-pressed to ensure their livestock get enough nutrition especially through the harsh dry seasons. Most communities in this arid region are reported to be resource-challenged, thus the farmers struggle to buy in feed supplements and necessary commercial preventative and curative chemicals to fight disease [1]. Livestock would survive on the surrounding key browse plant species in the rangeland such as shrubs, tubers and noxious plants especially during severe dry periods [5]. However, some of the plants could be lethal to the animals. *N. brachypus*, like many other noxious plants, have bioactive properties that are awaiting proper understanding.

While *N. brachypus* exhibits a considerable variation in leaf shape, three distinct colour differences in flesh; white, light brown and dark brown (Additional file 1) were observed when preparing the tubers to feed the cattle (Zananwe-personal communication). The white tubers are soft and exude milky white substance and the brown tubers are more fibrous. Even though cattle feed on all tuber types of *N. brachypus*, cattle preferred the white tubers. Appropriate identification and characterization of plant materials is essential for their successful selections, domestication and conservation. Plants have many features that aid in their identification such as dimension, branch shape and area of development but one of the most defining features is their leaf [6]. The importance of leaf shape as a defining feature to distinguish species has been acknowledged by [7–10]. However, the effect of stage of development, environment and management practices have been cited as the major disadvantages for using morphological and biochemical markers [11].

Because morphological characteristics are considerably affected by the environmental factors [12], molecular markers provide an important tool for assessing the genetic variability and structure of natural populations [13]. The use of molecular techniques such as DNA barcoding, amplified fragment length polymorphism (AFLP), single nucleotide polymorphism (SNP) and random amplification of polymorphic DNA (RAPD) are increasingly being used in plant diversity studies [14, 15]. Specifically RAPDs are increasingly being used because they are simple, quick and require no prior information of the sequence. RAPDs also provide markers that can be used to identify and discriminate genotypes, in addition to providing a means for assessing phenotypic expression and phylogenetic associations in the germplasm under study. RAPD analysis has been used extensively for genetic characterization of cassava plant accessions [16], the medicinal plant *Bacopa monnieri* [17], *hibiscus*, [18], African yam bean [11], *Jacaranda decurrens* Cham. [19] jatropha [20], sweet potato [21, 22], citrus [23] and apples [24]. However, some of the problems with RAPD are related to reproducibility, designing appropriate primers and amplification of RAPD-PCR products.

To our knowledge, nothing has been reported on the genetic diversity within *N. brachypus* wild plant accessions. Current use of the plant as a drought mitigation feed and for veterinary purposes is purely based on indigenous knowledge from the local communities. The objective of this study was to establish the diversity of *N. brachypus* wild plant species through phenotypic and genetic characterization using morphological descriptors and Random Amplified Polymorphic DNA (RAPD) markers.

## Methods

### Investigation 1: Characterization of *Neorautanenia brachypus* based on leaf morphology

#### Leaf sample collection

**Fig. 1:**
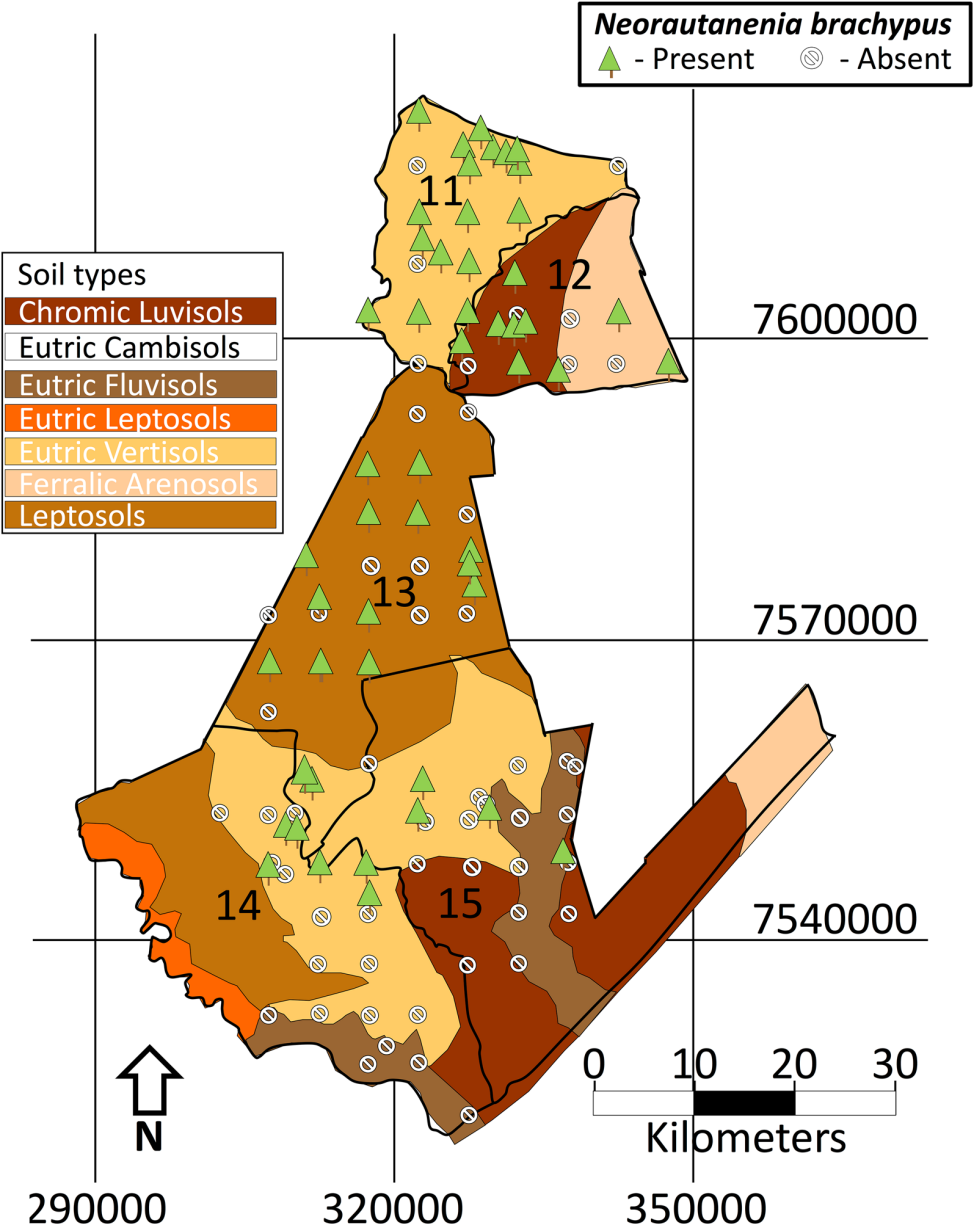
Distribution of *N. brachypus* in relation to soil type in the South-East Lowveld of Zimbabwe (Adapted with permission from Murungweni et al. [1])

A total of 100 leaf samples of *N. brachypus* were collected using judgmental sampling along a transect drive from wards 11–15 of Sengwe area, south of Gonarezhou National Park in Zimbabwe (Fig. 1). Judgmental or purposive sampling is a non-probability sampling technique where the researcher selects units to be sampled based on their knowledge and professional judgment. The sampling technique is employed especially when the desired population is uncommon and difficult to locate. Samples were selected based on perceived visual differences on leaf shape and stem colour. Fully expanded and non-senescent leaves were collected. The leaf samples were clearly labeled with (S) denoting the site from which the sample was collected and (PL) denoting the actual plant number sampled in the ascending order. Specific locations in terms of longitudinal and latitudinal coordinates were captured using a global positioning satellite system (Garmin eTrex 10). Additional information on the different soil type and surrounding vegetation was also collected. The leaf samples were classified based on leaf shape and maintained in a refrigerator at − 20 °C until they were used for DNA extraction.

#### Classification of *Neorautanenia brachypus* based on leaf shape

Morphological classification was done with respect to descriptors, leaf shape, leaf lobe type, leaf lobe numbers and shape of the central lobe according to [25] and tip shape, base shape and leaf margin [26]. The different features were recorded as binary data on the presence (one) or absence (zero) of features (Additional file 2). Photographs of the different leaf shapes were taken using a 16-megapixel digital camera for presentation.

A sample of 18 leaf biotypes was selected from the 96 accessions to represent the 14 visually clustered biotypes plus four whose leaves were not easily assigned to a group. From these 18, leaf shape was matched to their tuber pulp colour after cutting the tuber open. Pictures of leaf and the cut tubers were taken side by side. Classification was based on personal judgment basing on a tailor made sliding scale from one to six. The colour corresponded to the degree of whiteness of the tuber pulp and was scored one for more than 95% and for below 55%. The colour bars correspond to the degree of whiteness of the tuber pulp.

#### Statistical treatment of data

The analysis of morphological data was done using hierarchical clustering, a method that is widely used for grouping data over a variety of scales. The binary data was analysed using the Primer 7.0.13. Cluster analysis was performed on the basis of the genetic similarity matrix and the resulting similarity co-efficients were used for constructing a dendrogram using the unweighted pair group method with arithmetic average (UPGMA. Presence or absence data for morphological visually accessible traits was used. The similarities between matrices were based on Jacquard’s genetic similarity index. The selection of groups was based on number of clusters k-means using the Elbow method in the R package, where number of clusters is set at a level that explains most of the variability thus determining the threshold.

### Investigation 2: Classification of *Neorautanenia brachypus* based on RAPDs

#### Genomic DNA isolation from *N. brachypus* leaves

Molecular markers further characterized the morphologically different leaf groups that were described by hierarchical clustering above. Molecular characterization was important to confirm that the biotypes were indeed different considering that genes are a more precise measure of plant composition. DNA was extracted from young *N. brachypus* leaves according to the ZR Plant/Seed DNA MiniPrep ™ kit protocol (Catalog No. D6020—Zymo Research Corp). DNA was eluted in 60 µl of the DNA elution buffer. DNA quality was determined through gel electrophoresis in EZ-vision ^®^ (New England Bio labs) stained 1% agarose gel for sixty minutes at 100 volts in 1X Tris Borate EDTA (TBE).

#### PCR amplification

Forty three 10-mer random primers were sourced from Inqaba Biotechnology (Pty) Ltd South Africa. Primer selection was based on primers used in other RAPD marker studies for root and tuber crops ([11] and some randomly selected from Operon and the University of British Columbia pool [Operon Technologies, Alameda, Calif., USA) and University of British-Columbia, Canada (UBC primers)]. The lyophilized primers were reconstituted to a 10 µM solution by adding Tris Edta buffer according to synthesis report (Inqaba biotec). PCR conditions were optimized considering the Tm min/max of the different primers. The PCR reactions were conducted at a final volume of 25 µl, containing 5 µl DNA template, 4 µl primer (10 µM), 12.5 µl master mix (One Taq 2X master mix with standard buffer) and 3.5 µl nuclease free water. The PCR reaction was conducted using ARKTIK thermal cycler (Inqaba biotec). The thermal cycler was programmed for initial heat denaturation in one step of 1 min at 95 °C. Subsequent 45 cycles of denaturation for 1 min at 94 °C; annealing at 34.5 °C/36 °C for 1 min and extension at 72 °C for one minute. Final extension was at 72 °C for 10 min then holding at 4 °C infinite. The annealing temperature differed for the two groups of primer sets based on their differences in melting temperatures. Finally 10 µl of the PCR product was separated by gel electrophoresis in 1% agarose gel stained by EZ-vision ^®^ in gel stain in 1X TBE buffer for 90 min at 100 volts. PCR amplicon banding patterns were visualized in a gel documentation system (Infinity Vilber Lourmat—Inqaba Biotech) under ultra violet light. Images of the gel were captured on the documentation system. A total of 18 primers were selected from the initial 43 after a screening and PCR optimization process. The primers were screened for their ability to amplify genes of morphologically different leaf groups of *N. brachypus*.

#### RAPD scoring and data analysis

The amplification products (bands) for each lane were scored using the Vision Capt program on the gel documentation system (Infinity Vilber Lourmat). The bands were scored according to their molecular weight. The sizes of produced DNA fragments were estimated by comparison with the standard molecular marker 1 kb and 100 bp DNA ladder (New England BioLabs). The biotypes were scored for presence or absence of a particular DNA fragment size as one or zero respectively. The binary data produced from scoring the amplicon-banding pattern of the RAPD PCR were used for estimating genetic similarity coefficients. Bands were identified either as monomorphic or polymorphic. Monomorphic bands are those which are present in all individuals and polymorphic are unique ones that are absent in at least one individual not in any other (Additional file 3). Cluster analysis was performed using Jaccard’s genetic similarity index, and the resulting similarity co-efficient was used for constructing a dendrogram using the unweighted pair group method with arithmetic average (UPGMA) using Primer 7.0.13.

Polymorphic information content (PIC) values was calculated for each RAPD primer according to the formula:

$${\text{PIC}} = 1- {\text{R}}\left( {\text{Pij}} \right) 2,$$

where Pij is the frequency of the ith pattern revealed by the jth primer summed across all patterns revealed by the primers [27].

## Results

### Investigation 1: Characterization of *Neorautanenia brachypus* based on leaf morphology

**Fig. 2:**
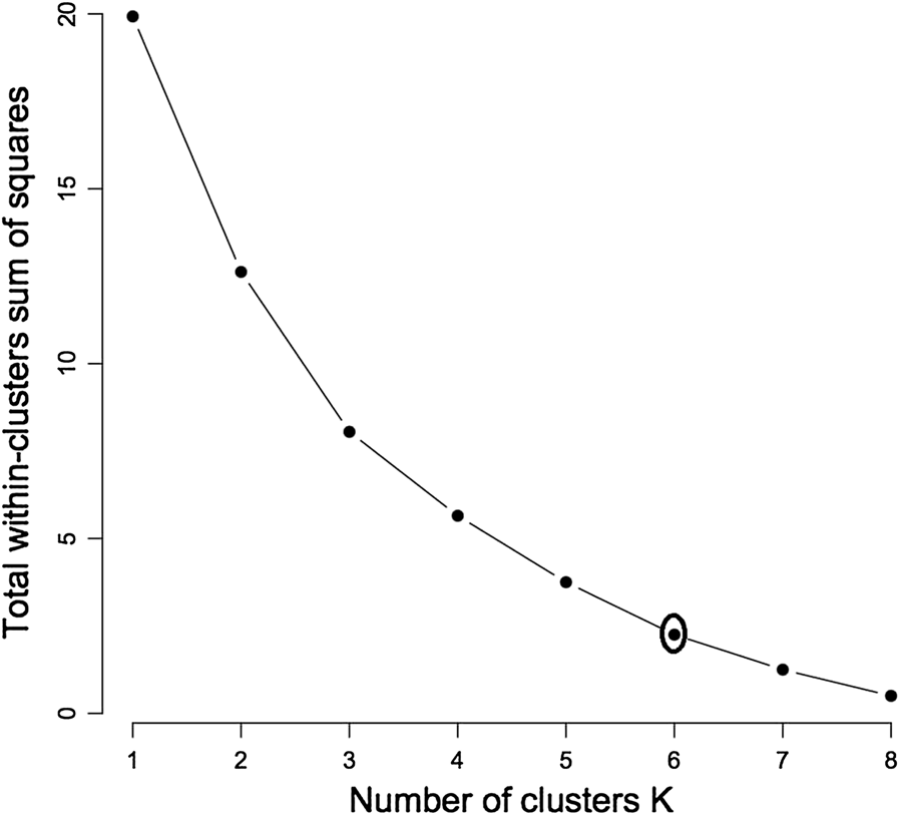
Cluster optimisation curve based on leaf morphology classified by leaf length (cm), width (mm) and length to width ratio at (K = 6)

**Fig. 3:**
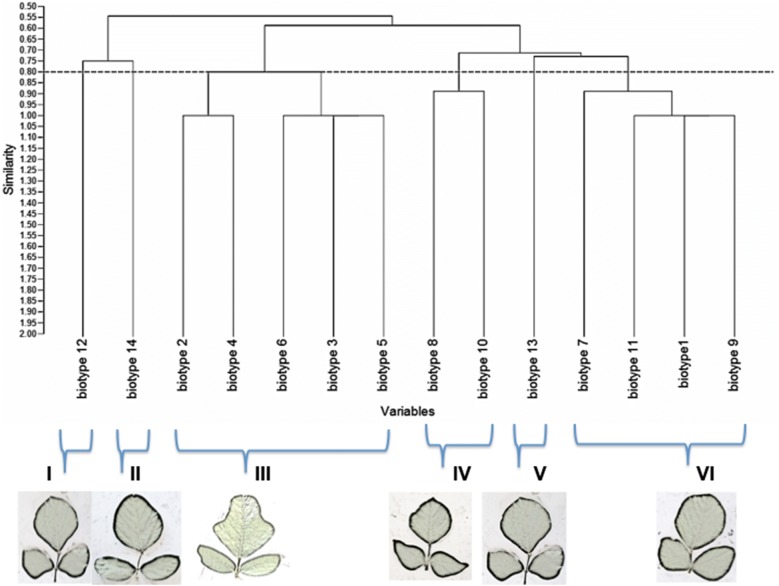
Dendrogram for *Neorautanenia brachypus* on the basis of the phenotypic characterization using hierarchical clustering at a threshold similarity level of 0.80

The findings from this study clearly show that there is some morphological diversity of leaves within the *N. brachypus* wild accession (Table 1). Initially, 14 morphological groups were identified by visual analysis of *N. brachypus* leaves. All the leaf samples displayed the arcuate venation with differences observed for leaf shape, tip shape and leaf margins. Biotype 12 constituted the largest proportion of leaf samples (27%) and the least proportion of 0.1% was found in biotype eight (Additional file 4: Appendix S1). Cluster optimization was performed to define the level at which most data is retained with variability well explained. The optimum number of clusters was at k = 6 (Fig. 2). When cluster analysis was performed based on leaf morphology (leaf lengths, widths and the length to width ratios), six leaf groupings were formed (Fig. 3) at a threshold of 0.80.

**Table 1:**
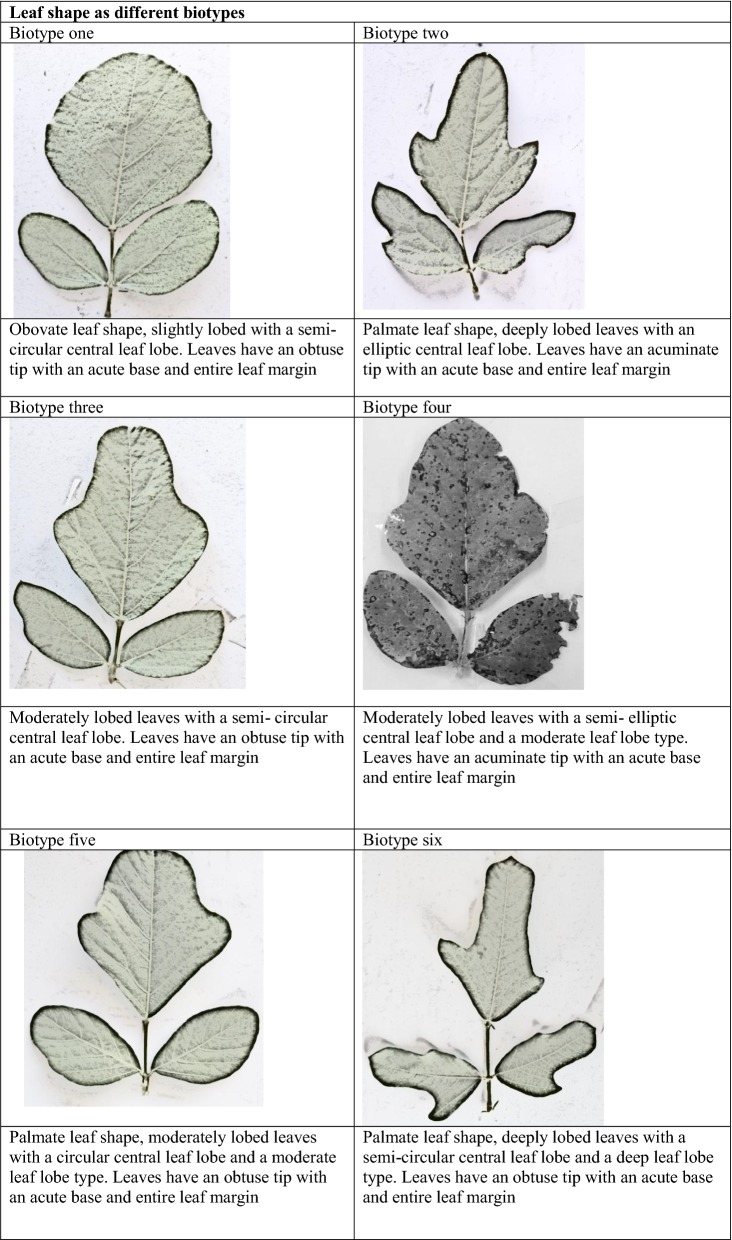

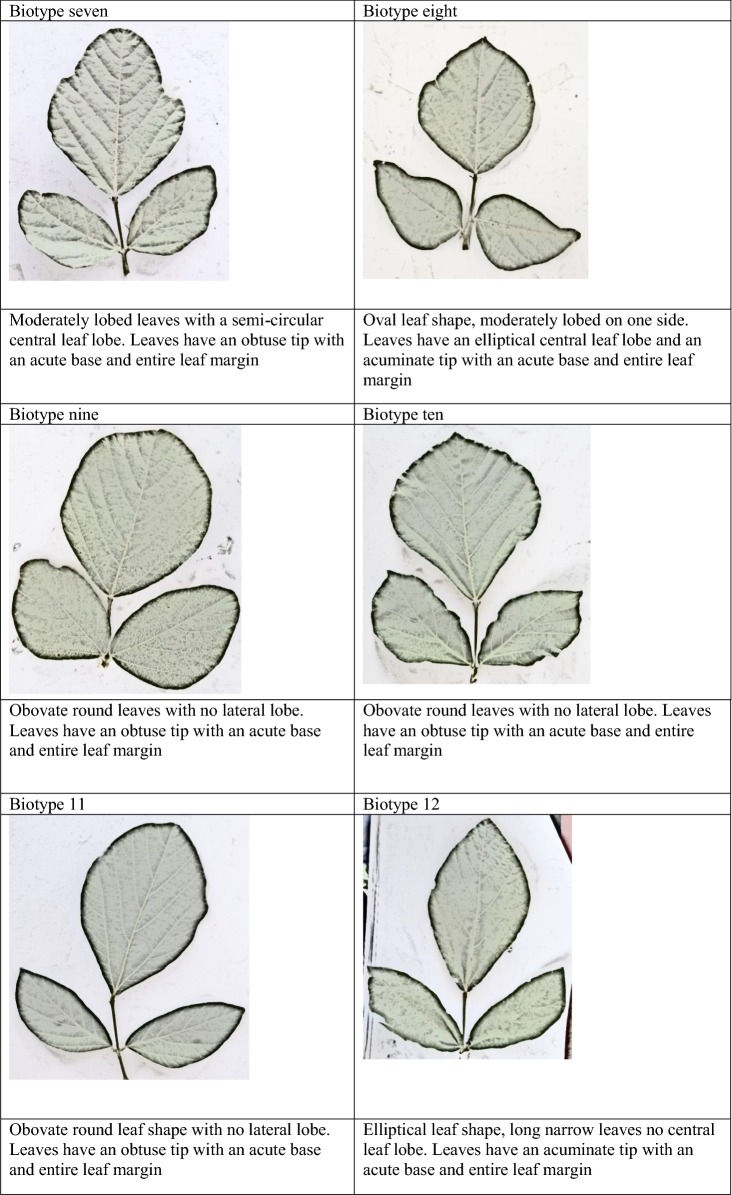

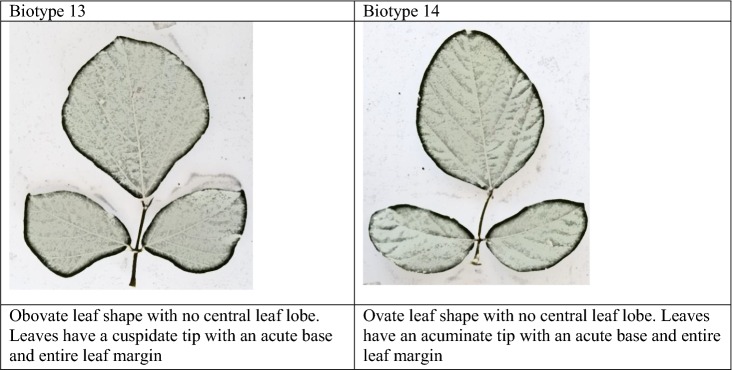
Leaf lamina shape variations among *Neorautanenia brachypus* leaf samples collected from five wards in Sengwe Chiredzi district, South-east Zimbabwe

#### Morphological descriptors used for separating *N. brachypus* plants by cluster analysis

Leaf shape traits separated the 14 selected accessions, representing the 14 visually grouped clusters, into five biotypes; the ovate, obovate, elliptical/diamond and hastate. Ovate leaf types are broad below the middle and roughly 2× as long as it is wide and can be viewed as egg shaped. While obovate leaf types are broadest above the middle and roughly 2× as long as it is wide (leaf shows the reverse of ovate). Triangular leaves with basal lobes are classified as hastate. Elliptical or diamond are leaves that had the broadest width in the middle and then taper off at the ends.

Leaf blade edge/margins was separated into three types; entire, lobate and undulate. Entire leaf edges are even and smooth throughout while lobate leaf edges are indented and undulate edges are wavy. Leaf veins are classified as arcuate where veins emerge from the central vein/mid rib in a sort of arc shape.

Tip shape was separated into three types; acuminate, cuspidate and obtuse. Acuminate tip shape are leaf blades with rounded shoulders leading to a pointed tip; cuspidate are leaf tip forming a short, narrow point. Obtuse leaves are narrow with a rounded tip.

**Fig. 4:**
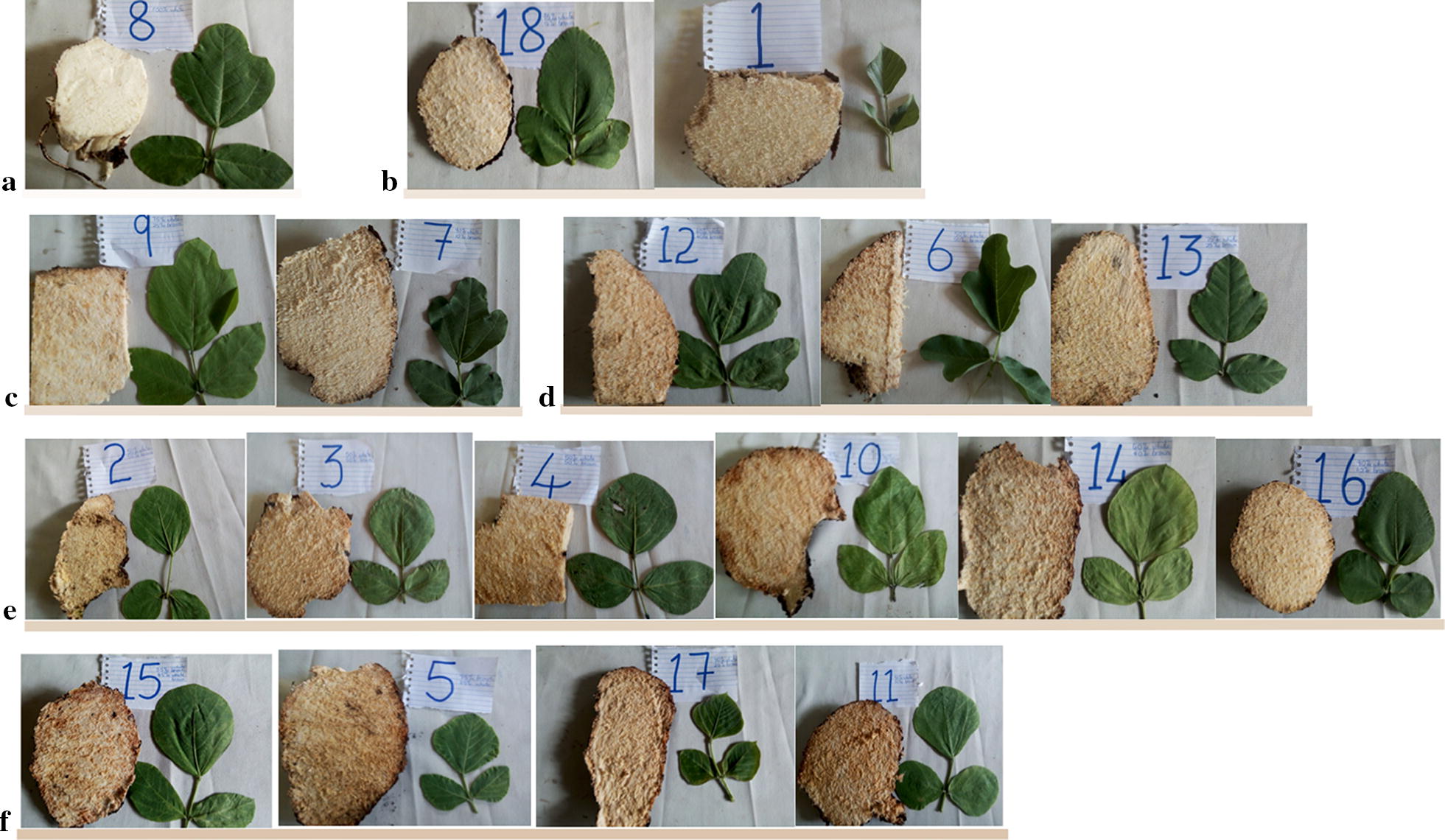
Flesh colour of *Neorautanenia brachypus* tubers in relation to leaf shape **a** 95% white, **b** 80% white, **c** 75% white, **d** 70% white, **e** 65% white, **f** 55%. Colour bars correspond to the degree of whiteness of the tuber pulp. The numbers on the pictures depict sample number on collection in the wild

The results show that there is some relationship between *N. brachypus* flesh colour and leaf shape (Fig. 4). It was observed that all 18 the leaf samples biotypes selected had more than 50% degree of whitening. The colour observation tended to cluster the lobed leaves together as shown by (c) and (d) groups.

### Investigation 2: Classification of *Neorautanenia brachypus* based on RAPDS

**Fig. 5:**
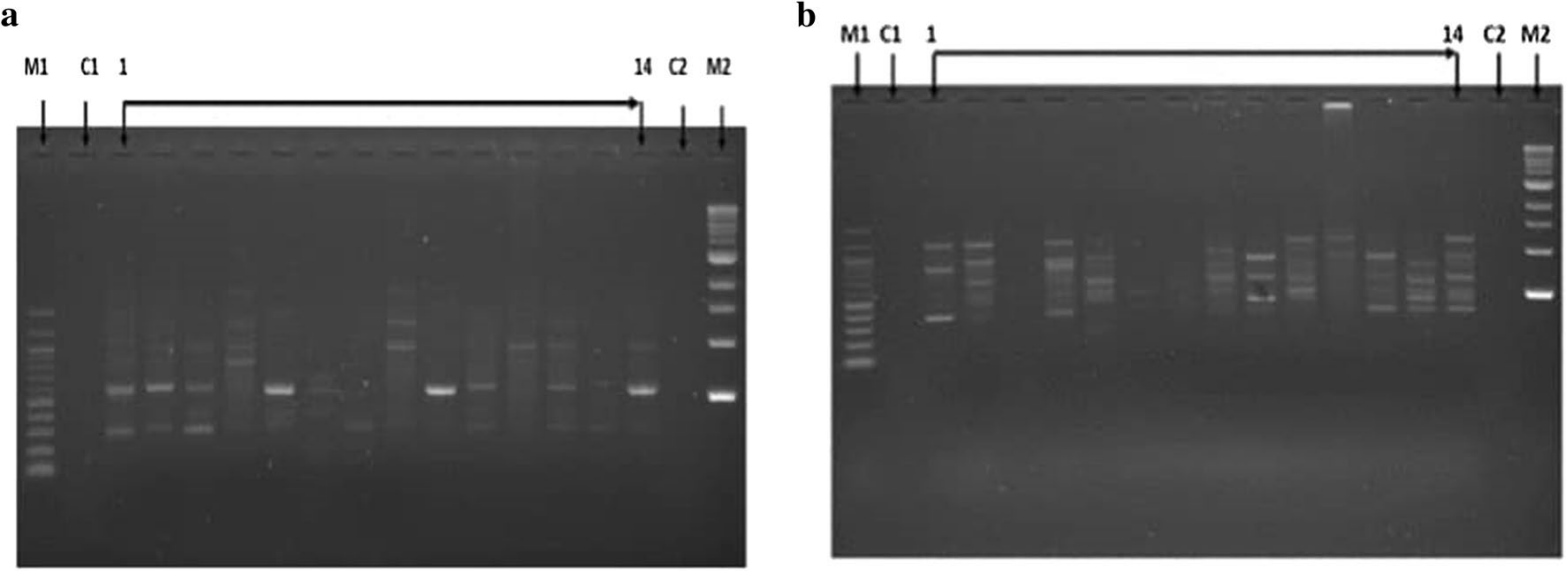
Profiles for RAPD bands obtained with the primers UBC 740 (**a**) and UBC 738 (**b**) for *Neorautanenia brachypus* wild accessions: M1 and M2 are 100 bp and 1 kb DNA markers respectively, C1 and C2 is the control. 1–14 indicate biotypes 1 to 14 as described in Table 1

The results from the study shows that the primers produced polymorphic RAPD fragment patterns. Figure 5 shows typical example of DNA polymorphism as detected for the Operon primers UBC 740 (a) and UBC 738 (b) respectively.

The selected 18 primers produced a total of 368 bands for the 14 biotypes with 77.5% of the genes showing polymorphism (Table 2). The number of bands produced per primer ranged from four (OPF-02) and 44 (UBC-746). The average number of bands per primer was 17.52 while average number of polymorphic bands was 13.95. The primer OPAD-9 gave the lowest percentage of polymorphism (16.7) while primers OPAB-17, OPAL -11, OPN-11 and UBC- 745 gave the highest percentage of polymorphism (100). The range of PIC values of the primers were 0.1327 (OPE-03) to 0.1873 (OPL-03) with an average of 0.157 (Table 2).

**Table 2:** Sequence of 18 RAPD-PCR primers, total number of scored bands, number of polymorphic bands, percentage polymorphism and polymorphic information content of *Neorautanenia brachypus* leaf samples

Primer	Primer sequence 5′ 3′	Total number of bands	Number of polymorphic bands	Percentage polymorphism	Polymorphic information content (PIC)
OPE-03	CCAGATGCAC	8	6	75.00	0.1327
OPL-03	CCAGCAGCTT	21	18	85.71	0.1873
OPAS-14	TCGCAGCGTT	6	4	66.67	0.1429
OPAB-17	TCGCATCCAG	11	11	100.00	0.1748
OPN-11	TCGCCGCAAA	8	8	100.00	0.1451
OPAL-14	TCGCTCCGTT	11	11	100.00	0.1497
UBC-434	TCGCTAGTCC	11	10	90.91	0.1439
UBC-737	GGTGGGTGTG	29	26	89.66	0.1534
UBC-738	GGTGGGTGGT	40	34	85.00	0.1747
UBC-742	CCTCCCTCCT	11	10	90.91	0.1327
UBC-743	CCACCCCAC	24	13	54.17	0.1622
UBC-744	CCACCCACCA	13	12	92.31	0.1439
UBC-745	GGGAAGAGGG	14	14	100.00	0.1514
UBC-746	GGGTGTTGGG	44	37	84.09	0.1487
UBC-740	GGAGGGAGGA	28	18	64.29	0.1851
OPAY-10	CAAGGCCCCT	22	14	63.64	0.1681
OPAY-11	ACGCGCCTTC	29	22	75.86	0.1727
OPAY-12	CTGTCGGCGT	21	18	85.71	0.1577
Total		368	293	–	
Average		17.52	13.95	77.51	0.157

**Fig. 6:**
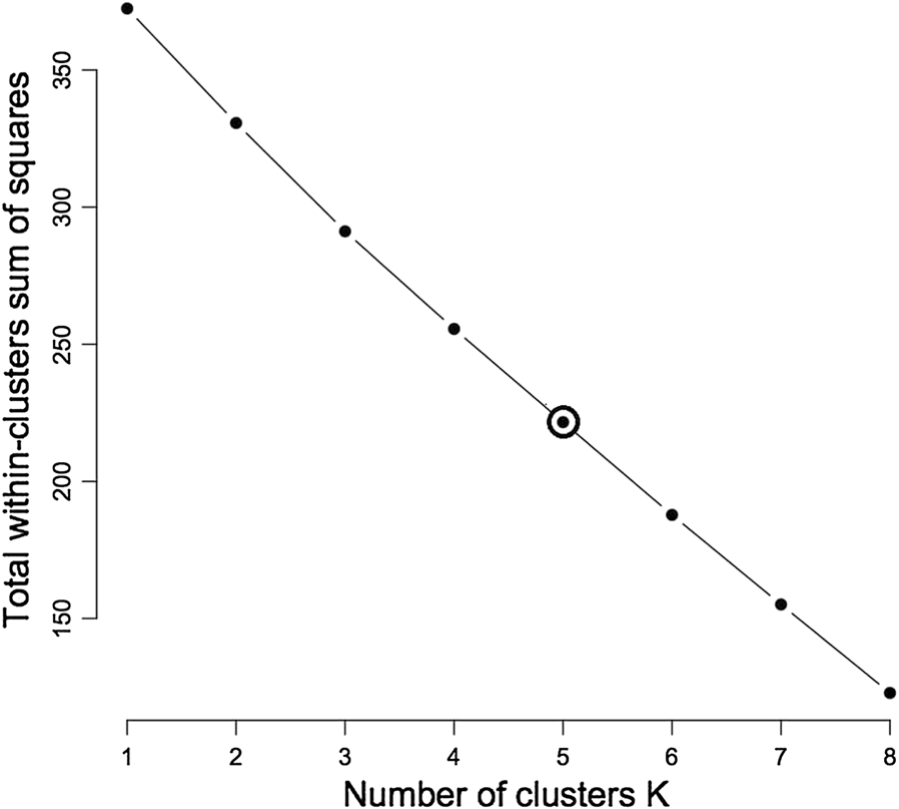
Cluster optimisation curve for *Neorautanenia brachypus* biotypes classified using RAPDs at best fit (k = 5)

**Fig. 7:**
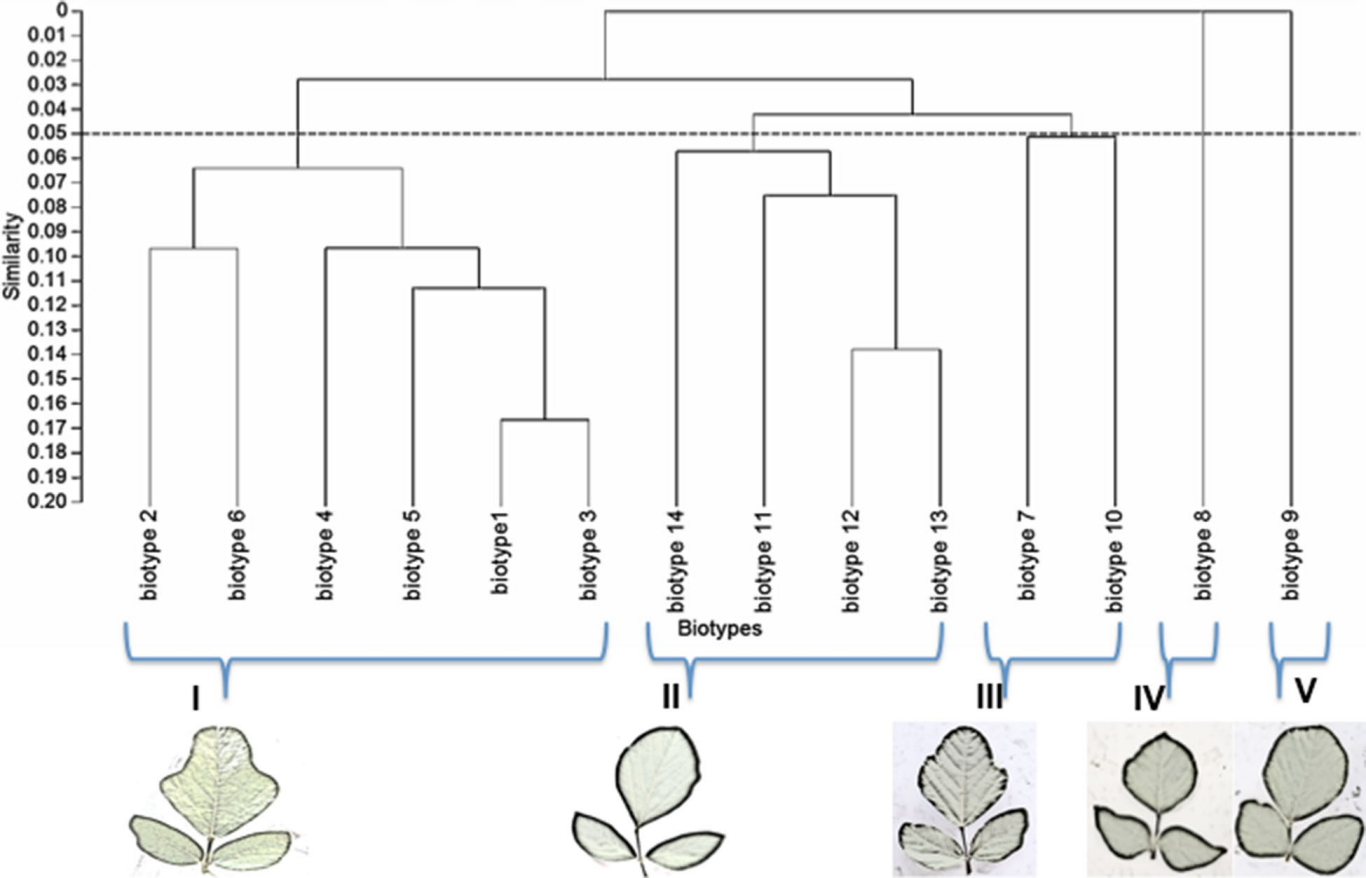
Dendrogram for *Neorautanenia brachypus* on the basis of combined RAPD profiles using hierarchical clustering at a threshold similarity distance level of 0.05

The cluster optimization was performed and was fit at k = 5 (Fig. 6). The dendrogram based on RAPD primers placed the 14 biotypes into five distinct clusters, I, II, III, IV and V (Fig. 7) with cluster grouping determined at threshold 0.05. Leaves in cluster I had a common feature of being lobed except biotype 1 as previously explained by leaf morphology. Cluster II had their leaf tip shape, base shape and entire margin in common except biotype 13. The third cluster had nothing in common. The fourth and fifth clusters were outliers, thus two independent groups constituting one biotype each.

**Fig. 8:**
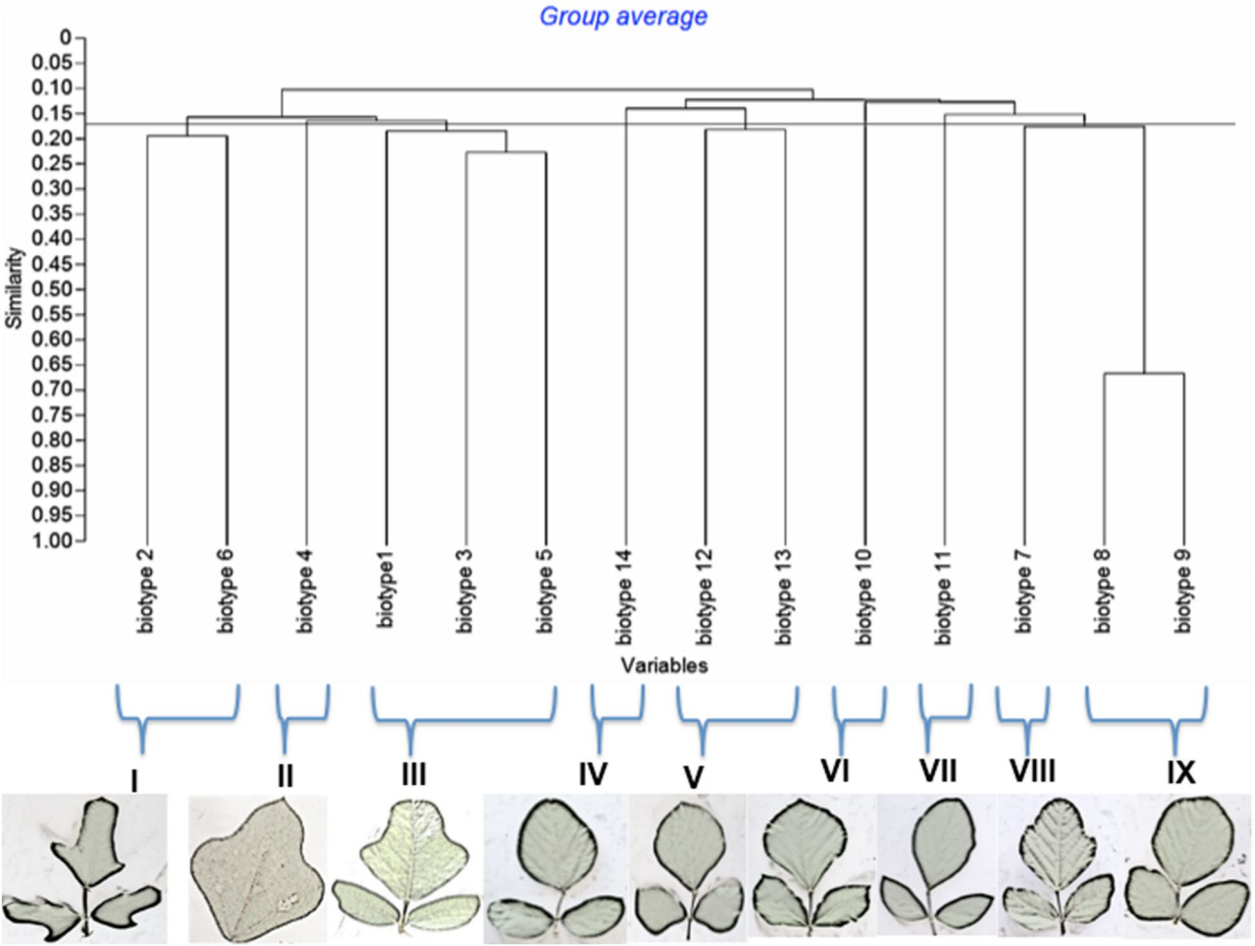
Dendrogram for *Neorautanenia brachypus* on the basis of combined phenotypic characterization and RAPD profiles using hierarchical clustering at a threshold similarity level of 0.16

**Fig. 9:**
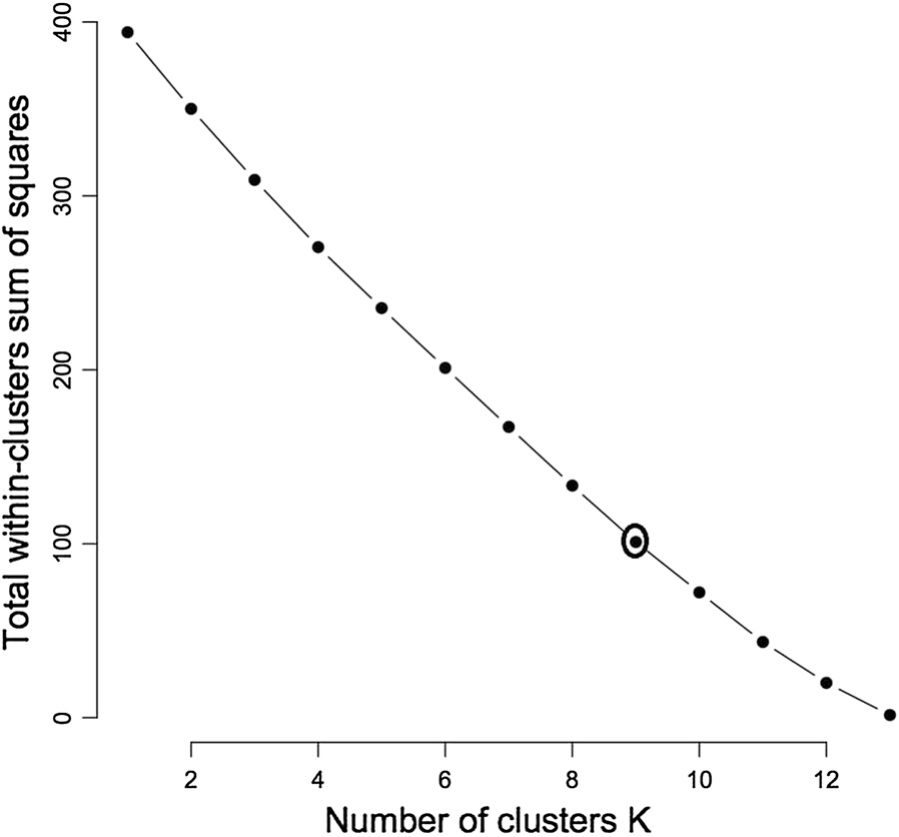
Cluster optimisation curve for *Neorautanenia brachypus* biotypes on the basis of combined phenotypic characterization and RAPD profiles at threshold (k = 9)

A combination of both morphological and molecular characterizatics placed the biotypes into nine distinct clusters (Fig. 8) after cluster optimization at k = 9 (Fig. 9). The combination maintained biotypes 2 and 6 and biotypes 3 and 5 as in morphology clustering.

## Discussion

This is the first study to use plant morphological characteristics and RAPD molecular markers to examine the genetic variability of *N. brachypus* wild population. The findings from this study show that there is both morphological and genetic diversity within the *N. brachypus* wild accession. Both the morphological and genetic differences substantiate observations by local farmers on tuber colour, quality and animal preferences as pointed out by Zanamwe (personal communications). However, some distinct variations noticeable by leaf shape could not be verified through molecular work as they were placed in different clusters by RAPDs. This confirms arguments by [28], who pointed out that, in general, the association of genetic variation with environmental conditions can be limited by natural selection and local gene dispersal. However, studies by [29] indicate that land use types significantly influences the structural and compositional attributes of vegetation. This finding supports work by [1] who identified that *N. brachypus* is widely distributed in cultivated fields.

Since leaves carry out photosynthesis that is important to plant growth and survival, variation in their shape could reflect natural selection for their function. A number of theories proposed to explain leaf diversity include thermoregulation of leaves mostly in arid and hot environments, hydraulic constraints, patterns of leaf expansion, biomechanical constraints, adaptations to avoid herbivory and adaptations to optimise light interception [30]. The variation in *N. brachypus* leaves could explain its adaptation to the semi-arid dry region of Zimbabwe. Being an angiospermous plant, *N. brachypus* also reflects random variation within the context of its phylogenetic history [31]. The leaf morphological variations across the biotypes are testimony to the importance of the leaf organ as an adaptive structure [32]. The lack of a defined relationship between leaf shape and tuber flesh colour can be better explained by its genetic makeup, location of growing and year of growing and or time of harvesting as described by [33, 34] in Irish potatoes. The study identified that the presence of carotenoids and anthocyanins positively influences tuber flesh colour.

However, due to these restrictions in morphological variability, [17] cited that molecular tools provide valuable data on diversity through their ability to detect variation at the DNA level. High genetic diversity among fruits has been reported; [23] on citrus and [24] on apples using plant morphology and RAPD markers. In this study RAPD primers were also able to detect some genetic diversity among *N. brachypus* wild accessions. The PIC values for 18 primers were on average 0.157 indicating their high discriminatory power. This PIC value is much lower than the maximum value given by [35] of 0.5 for dominant markers.

The combination of phenotypic characterization and RAPDs increased the resolution of identifying diversity as shown by the higher cophenetic correlation coefficient of 0.9316 and a cluster number of 9. However, there were some discrepancies existing between the morphological indicators and molecular indicators. Interestingly, morphological classification placed biotype two and biotype six in different groups but they were placed in one cluster with RAPDs. These discrepancies may be related to genotypes and the selection of RAPD primers. This lack of correlation between morphological traits and molecular markers could be explained by several factors as cited by [36]. The selected primers could not have covered vast area of *N. brachypus* genome or morphological variation could have been strongly influenced by environmental conditions or maybe the morphological similarities observed might be due to different combinations of alleles producing similar phenotypes. Cluster analysis with RAPDs narrowed down biotype groupings from ten to five. Different clustering of genotypes using plant morphology and RAPD polymorphism were previously reported from [24] on citrus, [17] on the medicinal plant *Bacopa monnieri* (L.) and [19] on *Jacaranda decurrens Cham.* This justifies arguments by [11] that morphological characters may not be obvious at all stages of plant development and appearance may be affected by environment.

## Conclusions

Findings of the present study reveal that *N. brachypus* germplasm presented some high diversity based on both phenotypic and RAPD-PCR assessment approaches. However, the results of both techniques are slightly different in comparison of their efficiency. The variability shows that RAPD markers are an ideal technique as they give more accurate assessments with a high level of precision as compared to plant morphology. However, it can be concluded that the techniques are more effective when used in combination since molecular work generally follows morphological characterisation. On the basis of results of this study, future studies on genetic diversity, using other molecular markers is possible for higher genetic resolution of the genome. The results of this study may be useful in establishing domestication and conservation strategies of *N. brachypus* in Zimbabwe. We are therefore recommending that selected biotypes be grown and monitored on their growth and developmental stages to ascertain their effects on the tuber colour and possibly composition, which is of preference to livestock.

## Additional files

**Additional file 1.** *Neorautanenia brachypus* tuber flesh colors. The file shows the main tuber flesh colors of sampled *Neorautanenia brachypus.* Three colour differences noted when tubers were cut across are white flesh, light brown flesh and dark brown flesh. Tubers of approximately same size were used as samples.

**Additional file 2.** Leaf morphological attributes scored. The file shows the measured as well as the scored leaf attributes of the descriptors for each sampled *Neorautanenia brachypus* plant accession. The columns are the descriptors while the rows are the plant accessions. Measurement and scoring of descriptors was done according to [22] and [23].

**Additional file 3.** Binary data presentation of the RAPD Polymorphic DNA fragments for the 14 morphologically distinguished biotypes. The file shows the binary score of each polymorphic DNA fragment produced by the RAPD banding pattern. The columns are the biotypes while the rows are the RAPD primers; indicating primer name and polymorphic DNA fragment size. The fragments are scored for each biotype as presence or absent; one and zero respectively.

**Additional file 4: Appendix S1.** GPS data from leaf collection sites. The file shows the geographical positions from which the *Neorautanenia brachypus* plants were collected. The coordinates shows the exact sites were plant specimens were collected in the South Eastern Lowveld of Zimbabwe. The selected sites were based on previous work by [4].

## Abbreviations

RAPD: Random Amplification of Polymorphic DNA
UPGMA: unweighted pair group method with arithmetic average
AFLP: amplified fragment length polymorphism
SNP: single nucleotide polymorphism
CP: cophenetic correlation coefficient
PIC: polymorphic information content
