# Supplementary figures and images for: Phenotypic and genetic characterisation revealed the existence of several biotypes within the Neorautanenia brachypus (Harms) C.A. wild accessions in South East Lowveld, Zimbabwe
Source: BMC Ecol. 2019 Mar 12;19:13. doi: 10.1186/s12898-019-0229-9 (PMC6417035; doi:10.1186/s12898-019-0229-9)

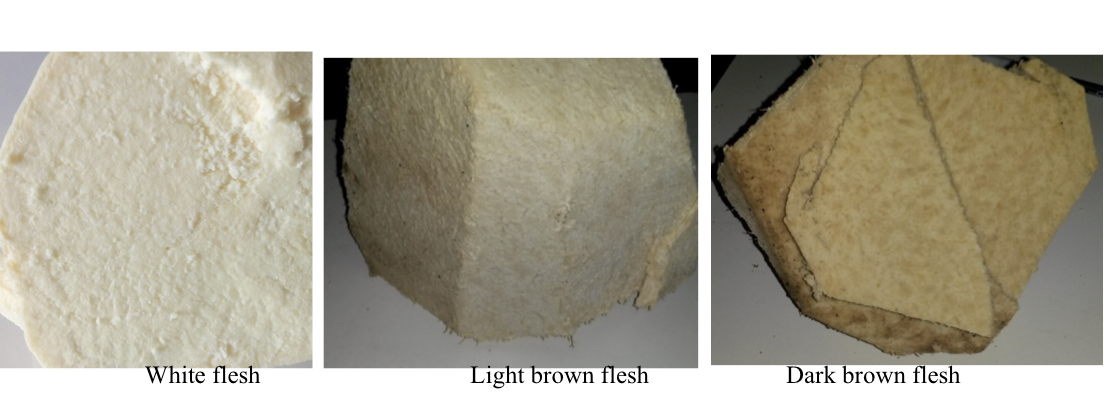

Supplement: Supplementary file 1 — Additional file 1. Neorautanenia brachypus tuber flesh colors. The file shows the main tuber flesh colors of sampled Neorautanenia brachypus. Three colour differences noted when tubers were cut across are white flesh, light brown flesh and dark brown flesh. Tubers of approximately same size were used as samples. [file 12898_2019_229_MOESM1_ESM.png]
