# Supplementary material for: Phenotypic and genetic characterisation revealed the existence of several biotypes within the Neorautanenia brachypus (Harms) C.A. wild accessions in South East Lowveld, Zimbabwe
Source: BMC Ecol. 2019 Mar 12;19:13. doi: 10.1186/s12898-019-0229-9 (PMC6417035; doi:10.1186/s12898-019-0229-9)
Supplement: Supplementary file 4 — Additional file 4: Appendix S1. GPS data from leaf collection sites. The file shows the geographical positions from which the Neorautanenia brachypus plants were collected. The coordinates shows the exact sites were plant specimens were collected in the South Eastern Lowveld of Zimbabwe. The selected sites were based on previous work by [4]. [file 12898_2019_229_MOESM4_ESM.docx]

Appendix S1: GPS data from leaf collection sites

| **Accession label** | **Latitude** | **Longitude** | **Leaf group** |
| --- | --- | --- | --- |
| S1/PL1 | 21°31. 193' | 031° 23.136' |  |
| S1/PL2 | 21°31. 183' | 031° 23.124' | 1 |
| S1/PL3 | 21°31. 190' | 031° 23.129' | 9 |
| S1/PL4 | 21°39. 818' | 031° 19.783' | 4 |
| S1/PL5 | 21°31. 201' | 031° 23.145' | 14 |
| S1/PL6 | 21°31. 205' | 031° 23.149' | 2 |
| S1/PL7 | 21°31. 220' | 031° 23.142' | 12 |
| S1/PL8 | 21°31. 232' | 031° 23.145' | 7 |
| S1/PL9 | 21°31. 260' | 031° 23.140' | 12 |
| S1/PL10 | 21°31. 268' | 031° 23.141' | 9 |
| S2/PL1 | 21°35. 808' | 031° 22.418' | 12 |
| S2/PL2 | 21°35. 807' | 031° 22.417' | 12 |
| S2/PL3 | 21°35. 808' | 031° 22.413' | 11 |
| S2/PL4 | 21°35. 807' | 031° 22.408' | 12 |
| S2/PL5 | 21°35. 807' | 031° 22. 409' | 14 |
| S3/PL1 | 21°37. 192' | 031° 21.312' | 7 |
| S3/PL2 | 21°37. 183' | 031° 21.315' | 12 |
| S3/PL3 | 21°37. 183' | 031° 21.315' | 12 |
| S3/PL4 | 21°37. 168' | 031° 21.300' | 12 |
| S3/PL5 | 21°37. 166' | 031° 21. 301' | 12 |
| S4/PL1 | 21°39. 753' | 031° 19.860' | 1 |
| S4/PL2 | 21°39. 764' | 031° 19.859' | 12 |
| S4/PL3 | 21°39. 767' | 031° 19.860' | 7 |
| S4/PL4 | 21°39. 768' | 031° 19.861' | 12 |
| S4/PL5 | 21°39. 786' | 031° 19.869' | 12 |
| S4/PL6 | 21°39. 785' | 031° 19.864' | 9 |
| S4/PL7 | 21°39. 776' | 031° 19.851' | 12 |
| S4/PL8 | 21°39. 818' | 031° 19.782' | 9 |
| S4/PL9 | 21°41. 841' | 031° 21.566' | 7 |
| S4/PL10 | 21°31. 195' | 031° 23.139' | 1 |
| S5/PL1 | 21°38. 653' | 031° 18.009' | 6 |
| S5/PL2 | 21°38. 645' | 031° 18.024' | 7 |
| S5/PL3 | 21°38. 646' | 031° 18.028' | 11 |
| S5/PL4 | 21°38. 650' | 031° 18.034' | 3 |
| S5/PL5 | 21°38. 651' | 031° 18.035' | 4 |
| S5/PL6 | 21°38. 645' | 031° 18.049' | 12 |
| S5/PL7 | 21°38. 651' | 031° 18.067' | 12 |
| S5/PL8 | 21°38. 678' | 031° 18.076' | 10 |
| S5/PL9 | 21°38. 680' | 031° 18.078' | 1 |
| S5/PL10 | 21°38. 683' | 031° 18.078' | 3 |
| S6/PL1 | 21°41. 911' | 031° 19.496' | 13 |
| S6/PL2 | 21°41. 921' | 031° 19.481' | 13 |
| S6/PL3 | 21°42. 017' | 031° 19.478' | 13 |
| S6/PL4 | 21°41. 920' | 031° 19.463' | 10 |
| S6/PL5 | 21°42. 006' | 031° 19.474' | 12 |
| S6/PL6 | 21°42. 006' | 031° 19.475' | 11 |
| S6/PL7 | 21°42. 039' | 031° 19.476' | 12 |
| S6/PL8 | 21°42. 043' | 031° 19.474' | 12 |
| S6/PL9 | 21°41. 920' | 031° 19.473' | 7 |
| S7/PL1 | 21°41. 546' | 031° 21.354' | 12 |
| S7/PL2 | 21°41. 538' | 031° 21.365' | 4 |
| S7/PL3 | 21°41. 534' | 031° 21.368' | 9 |
| S7/PL4 | 21°41. 521' | 031° 21.392' | 5 |
| S7/PL5 | 21°41. 511' | 031° 21.415' | 12 |
| S7/PL6 | 21°41. 502' | 031° 21.414' | 4 |
| S7/PL7 | 21°41. 502' | 031° 21.419' | 9 |
| S7/PL8 | 21°41. 502' | 031° 21.419' | 9 |
| S7/PL9 | 21°41. 498' | 031° 21.424' | 12 |
| S7/PL10 | 21°41. 497' | 031° 21.425' | 7 |
| S7/PL11 | 21°41. 476' | 031° 21.424' | 9 |
| S7/PL12 | 21°41. 479' | 031° 21.426' | 12 |
| S7/PL13 | 21°41. 477' | 031° 21.423' | 7 |
| S7/PL14 | 21°41. 474' | 031° 21.422' | 11 |
| S7/PL15 | 21°41. 478' | 031° 21.410' | 2 |
| S8/PL1 | 21°41. 876' | 031° 21.584' | 8 |
| S8/PL2 | 21°41. 874' | 031° 21.584' | 9 |
| S8/PL3 | 21°41. 870' | 031° 21.585' | 11 |
| S8/PL4 | 21°41. 847' | 031° 21.566' | 6 |
| S8/PL5 | 21°41. 849' | 031° 21.570' | 12 |
| S8/PL6 | 21°41. 843' | 031° 21.577' | 4 |
| S8/PL7 | 21°41. 840' | 031° 21.578' | 4 |
| S8/PL8 | 21°41. 866' | 031° 21.584' | 2 |
| S8/PL9 | 21°41. 868' | 031° 21.585' | 11 |
| S9/PL1 | 21°59. 715' | 031° 22.994' | 12 |
| S9/PL2 | 21°59. 706' | 031° 22.986' | 12 |
| S9/PL3 | 21°59. 711' | 031° 22.976' | 12 |
| S9/PL4 | 21°59. 712' | 031° 22.974' | 10 |
| S9/PL5 | 21°59. 710' | 031° 22. 973' | 1 |
| S9/PL6 | 21°59. 699' | 031° 22.971' | 4 |
| S9/PL7 | 21°59. 695' | 031° 22. 971' | 7 |
| S9/PL8 | 21°59. 692' | 031° 22. 971' | 12 |
| S10/PL1 | 21°59. 828' | 031° 23.029' | 9 |
| S10/PL2 | 21°59. 835' | 031° 23.028' | 9 |
| S10/PL3 | 21°59. 858' | 031° 23.026' | 9 |
| S10/PL4 | 21°59. 862' | 031° 23.027' | 12 |
| S10/PL5 | 21°59. 860' | 031° 23. 022' | 12 |
| S11/PL1 | 22°12. 607' | 031° 13.299' | 12 |
| S11/PL2 | 22°12. 606' | 031° 13. 299' | 9 |
| S12/PL1 | 22°09. 125' | 031° 11.180' | 12 |
| S12/PL2 | 22°09. 117' | 031° 11. 187' | 12 |
| S12/PL3 | 22°09. 116' | 031° 11.184' | 12 |
| S12/PL4 | 22°09. 112' | 031° 11. 165' | 1 |
| S12/PL5 | 22°09. 111' | 031° 11. 165' | 10 |
| S13/PL1 | 22°08. 340' | 031° 10.018' | 10 |
| S13/PL2 | 22°08. 338' | 031° 10. 020' | 1 |
| S13/PL3 | 22°08. 355' | 031° 10.031' | 9 |
| S13/PL4 | 22°08. 359' | 031° 10. 006' | 5 |
| S13/PL5 | 22°08. 360' | 031° 10. 011' | 12 |
| S13/PL6 | 22°08. 356' | 031° 10. 017' | 12 |
| S13/PL7 | 22°08. 365' | 031° 10. 031' | 7 |
